# Effects of rumen undegradable protein sources on nitrous oxide, methane and ammonia emission from the manure of feedlot-finished cattle

**DOI:** 10.1038/s41598-022-13100-9

**Published:** 2022-06-02

**Authors:** Larissa de Melo Coelho, Liziane de Figueiredo Brito, Juliana Duarte Messana, Abmael da Silva Cardoso, Geovany Macêdo Carvalho, Rodrigo de Nazaré Santos Torres, Roberta Souto Carlos, Euclides Braga Malheiros, Mara Cristina Pessôa da Cruz, Telma Teresinha Berchielli

**Affiliations:** 1 Department of Animal Science, Universidade Estadual Paulista (UNESP) - Faculdade de Ciências Agrarias E Veterinárias (FCAV), Jaboticabal, SP 14884-900 Brazil; 2 Department of Engineering and Exact Science, Universidade Estadual Paulista (UNESP) - Faculdade de Ciências Agrarias E Veterinárias (FCAV), Jaboticabal, SP 14884-900 Brazil; 3 Department of Soil Science, Universidade Estadual Paulista (UNESP) - Faculdade de Ciências Agrarias E Veterinárias (FCAV), Jaboticabal, SP 14884-900 Brazil

**Keywords:** Climate sciences, Environmental sciences

## Abstract

The effects of sources of rumen undegradable protein (RUP) in diets on methane (CH_4_), nitrous oxide (N_2_O) and ammonia (NH_3_) emissions from the manure of feedlot-finished cattle were evaluated. We hypothesized that the use of different RUP sources in diets would reduce N loss via urine and contribute to reduced N_2_O, CH_4_ and NH_3_ emissions to the environment. Nellore cattle received different diets (18 animals/treatment), including soybean meal (SM, RDP source), by-pass soybean meal (BSM, RUP source) and corn gluten meal (CGM, RUP source). The protein source did not affect the N and C concentration in urine, C concentration in feces, and N balance (P > 0.05). The RUP sources resulted in a higher N_2_O emission than the RDP source (P = 0.030), while BSM resulted in a higher N_2_O emission than CGM (P = 0.038) (SM = 633, BSM = 2521, and CGM = 1153 g ha^−2^ N–N_2_O); however, there were no differences in CH_4_ and NH_3_ emission (P > 0.05). In conclusion, the use of RUP in diets did not affect N excretion of beef cattle or CH_4_ and NH_3_ emission from manure, but increased N_2_O emission from the manure.

## Introduction

Finishing cattle in confinement feedlots enables the use of feed sources that are adequate for the animal’s requirements, which increases productivity and meat quality^1^. However, this system is responsible for a greater accumulation of manure, which contains several components such as N and organic materials^2^. These components may undergo transformation and serve as a source of emission of greenhouse gases (GHGs), such as nitrous oxide (N_2_O) and methane (CH_4_)^3–5^, as well as of ammonia (NH_3_)^6,7^. Greenhouse gas emissions contribute to global warming^8^, whereas NH_3_ volatilization harms human health^7,9^ and potentially increase GHG emissions as NH_3_ is a precursor for N_2_O generation^10^.

Nitrous oxide is emitted through the transformation of ammonium (NH_4_^+^) and nitrate (NO_3_^–^) in soil during nitrification, denitrification^11^, and nitrifier denitrification^12^ mediated by fungi, bacteria and archaea^13^. These processes are affected by precipitation, temperature and substrate availability^14,15^. The magnitude of gas emission from cattle manure depends on the form and concentration of N^16^. Therefore, the reduction of N loss via ruminant excreta, specifically of N in the form of urea, is relevant to mitigate N_2_O emission, since 70% of the N excreted by ruminants is in the form of urea, which releases NH_4_^+^ following hydrolysis^17^. In addition, microbial hydrolysis of urea results in NH_3_ emission^18^; thus, the reduction of N-urea from excreta might directly reduce NH_3_ emission^19^.

The amount of CH_4_ emitted from manure is small compared with the total amount of enteric CH_4_ produced by ruminants^20^. However, emission from manure in feedlots is relevant, because large volumes of manure can result in higher CH_4_ emission^21^. Nitrogen and C content^22^, moisture, and temperature^23^, are the major modulators of CH_4_ emissions. Strategies aimed at increasing the efficiency of N use, resulting in lower N excretion, can modify the CN ratio of manure, which is an important factor responsible for the reduction of CH_4_ emission^24^. The high CN ratio can promote the growth of populations of methanogenic archaea that are able to meet their protein requirements and therefore not react with the remaining carbon content of the substrate, resulting in low production of CH_4_^25^. Thus, reducing nutrient excretion by animals may serve as a strategy to mitigate CH_4_ emission from manure.

Optimizing the use of N by ruminants can reduce N loss through urine and, therefore, minimize NH_3_^7^, and N_2_O emission from manure^26^. Reducing the amount of rumen degradable protein (RDP) and increasing the amount of ruminal undegradable protein (RUP) in diets may increase overall N efficiency and enable adequate supply of metabolizable protein (PM) to reach the small intestine^27^. Thus, we hypothesized that different sources of RUP in the diets would reduce N loss via urine and contribute to decreased N_2_O, CH_4_, and NH_3_ emissions to the environment. By modulating the diet in order to reduce N excretion, there is a possibility of impacting the production of enteric CH_4_^28^. However, in our study, the focus was intended to understand how the sources of RUP can affect the emission in the excreta, consequently, the emission of enteric CH_4_ was not measured. The evaluation in-situ will enable get more representative emissions from the feedlot environment. Therefore, the objective of the present study was to evaluate the effects of sources of RUP in diets on N_2_O, CH_4_ and NH_3_ emissions from manure of feedlot-finished Nellore and identify key driving variables that regulate the production of these gases.

## Results

### Characterization of animals’ excreta and N balance

There were no differences in the C and N content or C/N of the urine and fecal samples between the RUP and RDP sources (P > 0.05) (Table 1). Inclusion of CGM as a source of RUP in the diet increased N content (P = 0.012) but decreased the C/N in the fecal samples compared with the inclusion of BSM as a source of RUP (P = 0.009). However, there were no differences in the C/N of urine samples between the RUP and RDP sources (P = 0.632).

**Table 1:** Fecal and urinary N content and C and N balance of Nellore cattle fed with sources of rumen undegradable protein during the finishing phase in feedlots.

Variables	Treatments^1^			SEM^2^	*p* value	
	SM	BSM	CGM		RDP vs*.* RUP	BSM vs. CGM
**Chemical composition**						
Feces						
N, g kg^−1^ DM	33.6	33.2	34.9	0.5	0.444	0.012
C, g kg^−1^ DM	409.7	413.0	410.4	3.9	0.695	0.637
C/N	12.2	12.4	11.7	0.1	0.658	0.009
**Urine**						
N, g kg^−1^ DM	5.1	6.1	5.6	0.8	0.419	0.669
C, g kg^−1^ DM	8.2	10.8	8.8	1.5	0.382	0.186
C/N	1.5	1.8	1.4	0.1	0.632	< 0.001
**N balance**						
**N, g day**^**−1**^						
Intake	223.1	209.8	204.6	10.4	0.224	0.724
Fecal excretion	86.4	89.6	82.4	5.5	0.952	0.358
Urinary excretion	83.4	75.8	77.8	5.8	0.391	0.659
Total excretion	169.8	165.4	160.2	9.3	0.548	0.691
Total retention	53.3	44.3	44.4	5.4	0.194	0.995
**N, % intake**						
Fecal N	38.7	42.7	39.8	1.2	0.101	0.097
Urinary N	37.6	36.0	38.5	2.3	0.913	0.450
N retention	23.7	21.2	21.7	2.2	0.428	0.886
**N, % excretion**						
Urine	51.3	54.5	51.2	1.9	0.521	0.227
Feces	48.7	45.5	48.8	1.9	0.521	0.227

^1^SM = manure of animals fed soybean meal as a source of rumen degradable protein (RDP), BSM = manure of animals fed by-pass soybean meal as a source of rumen undegradable protein (RUP), CGM = manure of animals fed corn gluten meal as a source of RUP; ^2^SEM = standard error of the mean. Animal considered as an experimental unit (n = 9).

None of the three evaluated protein sources affected N consumption, fecal and urinary N excretion, total N excretion and total N retention (P > 0.05). There were no differences in fecal and urinary N excretion, N retention (% intake) or fecal and urinary N excretion (% excreted) among the three protein sources (P > 0.05).

### Gas emissions

**Figure 1:**
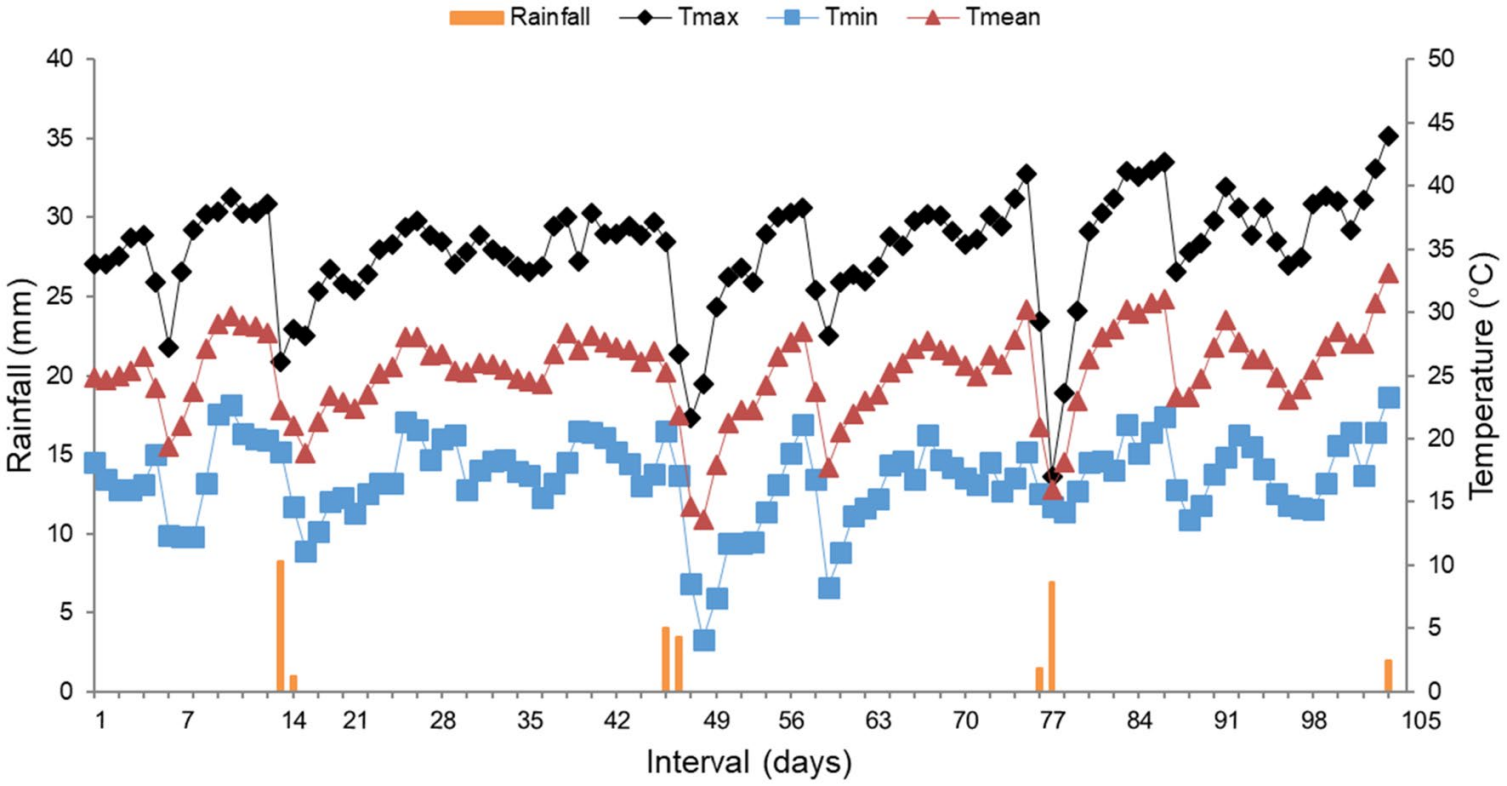
Daily rainfall and daily minimum (Tmin), daily, mean (Tmean) and daily maximum (Tmax) ambient temperature throughout the N_2_O and CH_4_ emission sampling period. Data were retrieved from the Agrometeorological Station, Department of Exact Sciences (FCAV/UNESP), located at 1 km from the experimental area.

Mean temperature during the N_2_O and CH_4_ emission sampling period was 20 °C; the lowest (3.3 °C) and highest (35.2 °C) temperatures were recorded close to sampling day 49 and on the last sampling day, respectively. Cumulative precipitation throughout the experimental period was 33.6 mm, occurring over 7 different days (Fig. 1).

**Figure 2:**
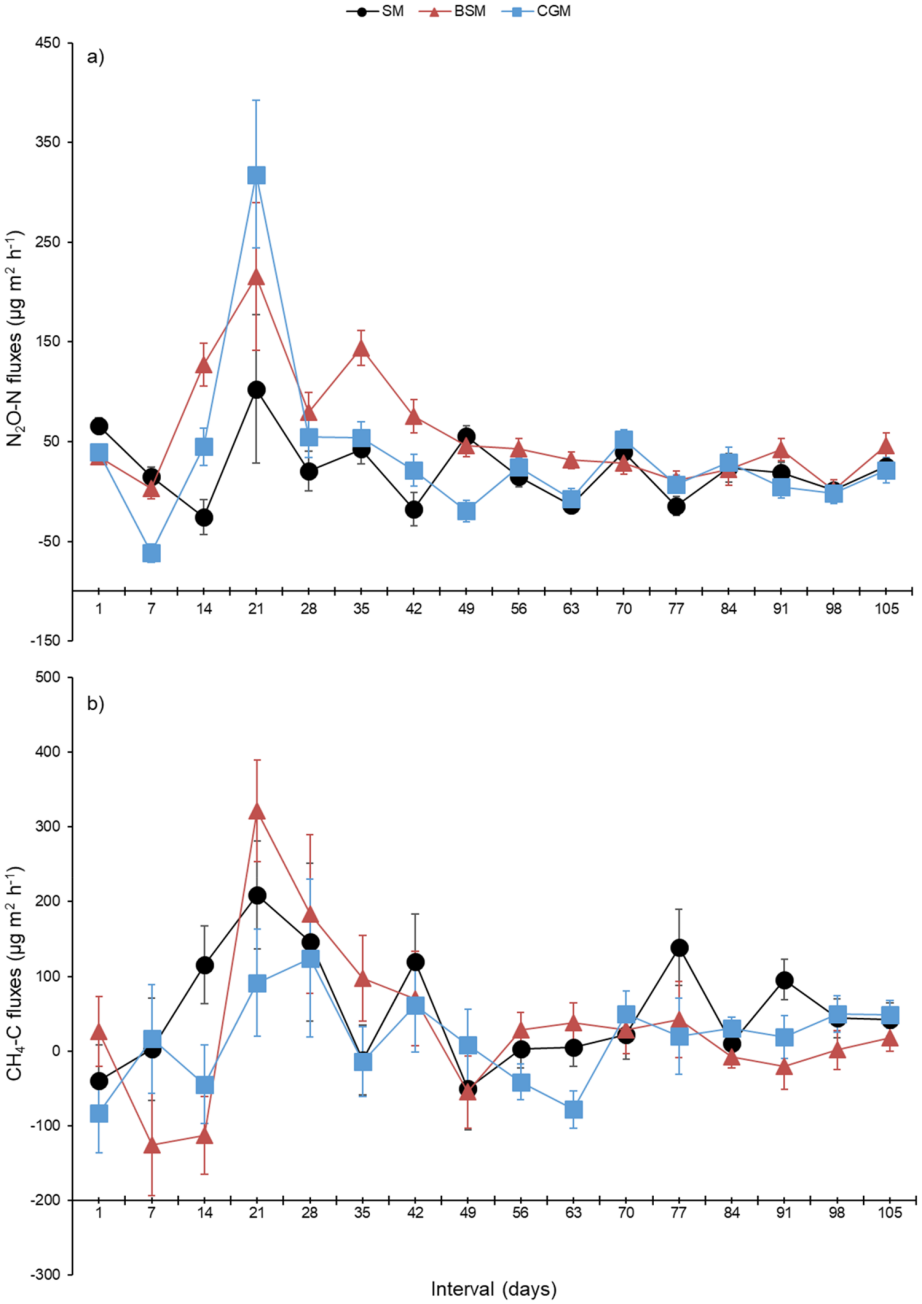
N_2_O and CH_4_ fluxes from the manure of Nellore cattle fed with sources of rumen undegradable protein during the finishing phase in feedlots. SM = manure of animals fed soybean meal as a source of rumen degradable protein (RDP), BSM = manure of animals fed by-pass soybean meal as a source of rumen undegradable protein (RUP), CGM = manure of animals fed gluten meal as a source of RUP. P-values for N_2_O (treatment = 0.003; time < 0.001; treatment × time interaction < 0.001) and CH_4_ (treatment = 0.165; time < 0.001; treatment × time interaction < 0.005). Chamber considered as an experimental unit (n = 9). The error bars representing standard error of the mean.

Daily mean N_2_O and CH_4_ fluxes varied from −62 to 318 µg N_2_O m^2^ h^−1^ and from −125 to 321 µg CH_4_ m^2^ h^−1^, respectively, during the experimental period (Fig. 2). Highest peak of N_2_O emission was observed in the 21st day, on all treatments. On the same day, an increase in CH_4_ fluxes was also observed. Differences in N_2_O and CH_4_ fluxes among treatments occurred in some days of evaluation and were not consistent along the studied period.

Protein sources did not affect cumulative CH_4_ emission from animal manure (P > 0.05) (Table 2). However, the manure of animals fed RUP sources resulted in a higher cumulative N_2_O emission than that of animals fed the RDP source (P = 0.030). Emissions from manure of cattle fed CGM were almost double and emissions from manure from cattle fed BSM (P = 0.038) were quadrupled compared to SM-fed cattle.

**Table 2:** Cumulative CH_4_ and N_2_O emissions from the manure of Nellore cattle fed with sources of rumen undegradable protein during the finishing phase in feedlots.

Variables, g ha^−1^	Treatments^1^			SEM^2^	*p* value	
	SM	BSM	CGM		RDP vs*.* RUP	BSM vs*.* CGM
CH_4_-C	1352	801	834	429	0.320	0.957
N_2_O-N	633	2521	1153	430	0.030	0.038

^1^SM = manure of animals fed soybean meal as a source of rumen degradable protein (RDP), BSM = manure of animals fed by-pass soybean meal as a source of rumen undegradable protein (RUP), CGM = manure of animals fed corn gluten meal as a source of RUP; ^2^SEM = standard error of the mean. Chamber considered as an experimental unit (n = 9). The cumulative values refer to 112 days of feedlot.

**Figure 3:**
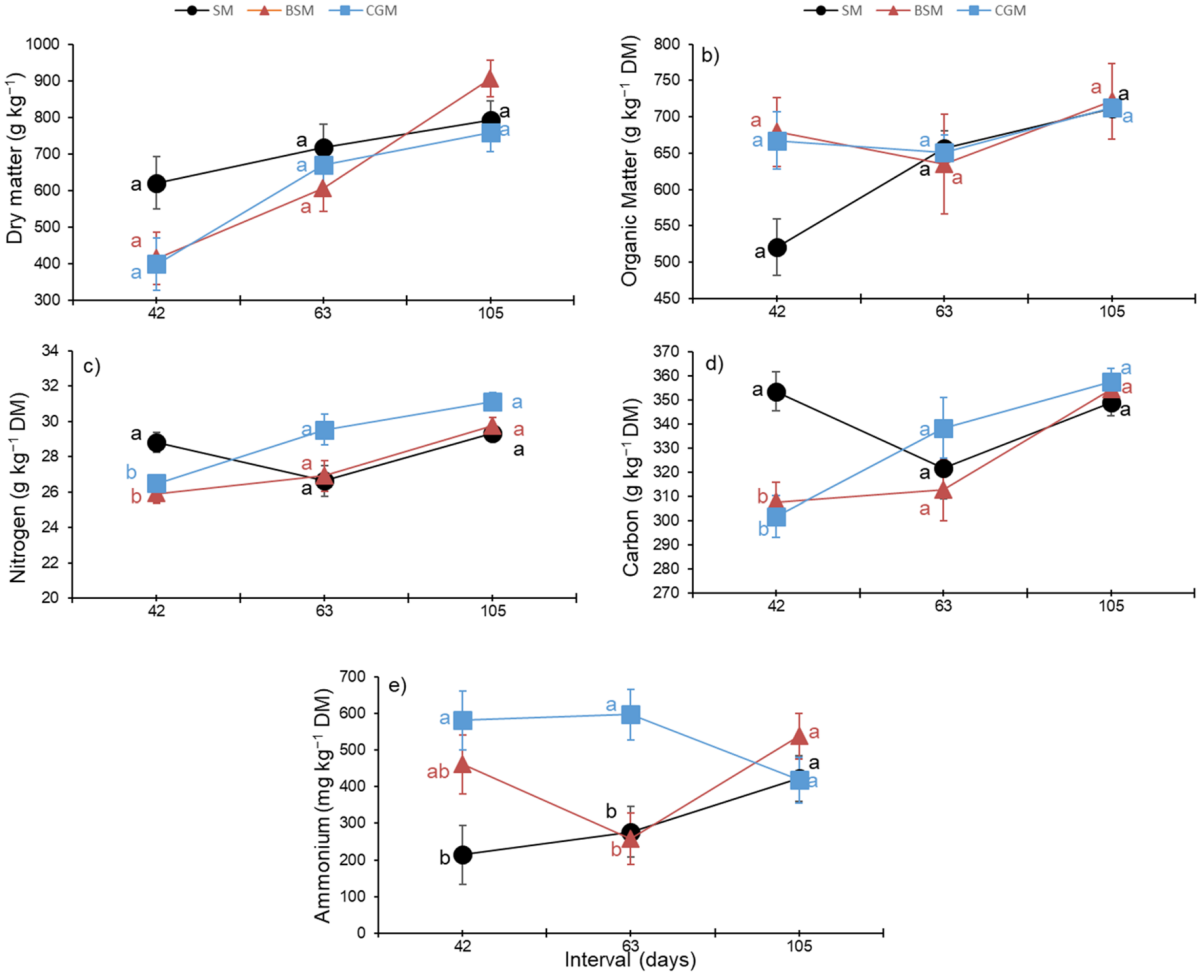
Dry matter, organic matter, N, C and NH_4_^+^ content of the manure, deposited in the soil, of Nellore cattle fed with rumen undegradable protein source during the finishing phase in feedlot. SM = manure of animals fed soybean meal as a source of rumen degradable protein (RDP), BSM = manure of animals fed by-pass soybean meal as a source of rumen undegradable protein (RUP), CGM = manure of animals fed corn gluten meal as a source of RUP. Different letters represent significant differences by Tukey's Test (P ≤ 0.05) within the treatment vs time interaction. Chamber considered as an experimental unit (n = 9). The error bars representing standard error of the mean.

An interaction between sampling time and protein source was observed for DM, OM, N, C and NH_4_^+^ (Table 3, Fig. 3). The manure of animals fed CGM presented a lower N content and higher NH_4_^+^ than that of animals fed SM on day 42 (P < 0.001), while on day 63 higher values of N and NH_4_^+^ were observed for the manure of animals fed CGM in relation to BSM (P = 0.002 and P = 0.010 respectively) and SM (P = 0.004 and P < 0.001, respectively). The manure of animals fed SM showed a higher C content than that of animals fed source of RUP on day 42 (P = 0.001). The manure of animals fed SM showed a higher C/N than that of animals fed RUP (P = 0.001). Nitrate content of the analyzed samples was not detectable.

**Table 3:** Chemical composition of the manure, deposited in the soil, of Nellore cattle fed with sources of rumen undegradable protein during the finishing phase in feedlot.

Var^1^	Treatment^2^			SEM	*p* value			
	SM	BSM	CGM		RDP vs*.* RUP	BSM vs*.* CGM	T^4^	TR vs*.* T^5^
DM	710	642	608	42	0.204	0.260	0.239	0.030
OM	629	678	677	15	0.231	0.036	0.057	0.044
N	28.4	27.5	29.2	0.4	0.039	0.167	0.050	< 0.001
C	341	324	332	6	0.913	0.077	0.201	< 0.001
C/N	12.1	11.8	11.5	0.1	0.001	0.061	0.002	0.297
NH_4_^+^	304	400	532	39	0.001	0.1026	0.001	< 0.001
NO_3_^−^	–	–	–	–	–	–	–	–

^1^DM = dry matter (g kg^−1^), OM = organic matter (g kg^−1^), N = nitrogen (g kg^−1^), C = carbon (g kg^−1^), NH_4_^+^ ammonium (mg kg^−1^); ^2^SM = manure of animals fed soybean meal as a source of rumen degradable protein (RDP), BSM = manure of animals fed by-pass soybean meal as a source of rumen undegradable protein (RUP), CGM = manure of animals fed corn gluten meal as a source of RUP.;^3^SEM = standard error of the mean; ^4^ T = time; ^5^Interaction TR (treatments = SM, BSM and CGM) × T (time). Chamber considered as an experimental unit (n = 9).

There were no correlations of manure gases (N_2_O and CH_4_) emissions with N, C, C/N ratio, DM, OM, and NH_4_^+^ (P > 0.05) (Table 4). Nitrogen was positively correlated with C (P < 0.001) and OM (P < 0.002). Carbon was positively correlated with C/N ratio (P < 0.001). Ammonium was positively correlated with OM (P = 0.045).

**Table 4:** Pearson’s correlation coefficients between explanatory variables during the evaluated period.

Variables^1^	CH_4_-C	N	C	C/N	DM	OM	NH_4_^+^
N_2_O-N	0.018	−0.104	−0.103	−0.015	−0.014	−0.012	−0.035
CH_4_-C		−0.016	0.092	0.210	−0.074	−0.081	−0.058
N			0.851*	−0.137	0.207	0.329*	0.062
C				0.401*	0.126	0.355	0.018
C/N					−0.133	0.092	−0.070
DM						−0.183	0.115
OM							0.224*

*Represents a statistical significance (P ≤ 0.05) for the coefficients of correlation. Analyses were carried out using data from all evaluated days. ^1^DM = dry matter, OM = organic matter, NH_4_^+^ = ammonium.

A positive correlation was observed between CH_4_ and C/N ratio on day 42 (P = 0.025), and between CH_4_ and NH_4_^+^ on day 63 (P = 0.001). On day 105, N_2_O was positively correlated with DM (P = 0.018) and NH_4_^+^ (P = 0.008) (Table 5).

**Table 5:** Pearson’s correlation coefficients between explanatory variables on each sampling day.

Variables^1^	N	C	C/N	DM	OM	NH_4_^+^
	**Day 42**					
N_2_O-N	0.047	0.022	−0.022	0.001	0.192	−0.122
CH_4_-C	0.051	0.297	0.430*	0.003	−0.152	0.014
	**Day 63**					
N_2_O-N	−0.197	−0.182	0.050	0.018	0.254	0.030
CH_4_-C	−0.037	0.081	0.252	−0.268	0.174	0.592*
	**Day 105**					
N_2_O-N	0.069	0.093	−0.004	0.449*	0.120	0.497*
CH_4_-C	0.155	0.202	−0.017	0.252	0.201	0.159

*Represents a statistical significance (P ≤ 0.05) for the coefficients of correlation. ^1^DM = dry matter, OM = organic matter, NH_4_^+^ = ammonium.

### NH_3_ emission

**Figure 4:**
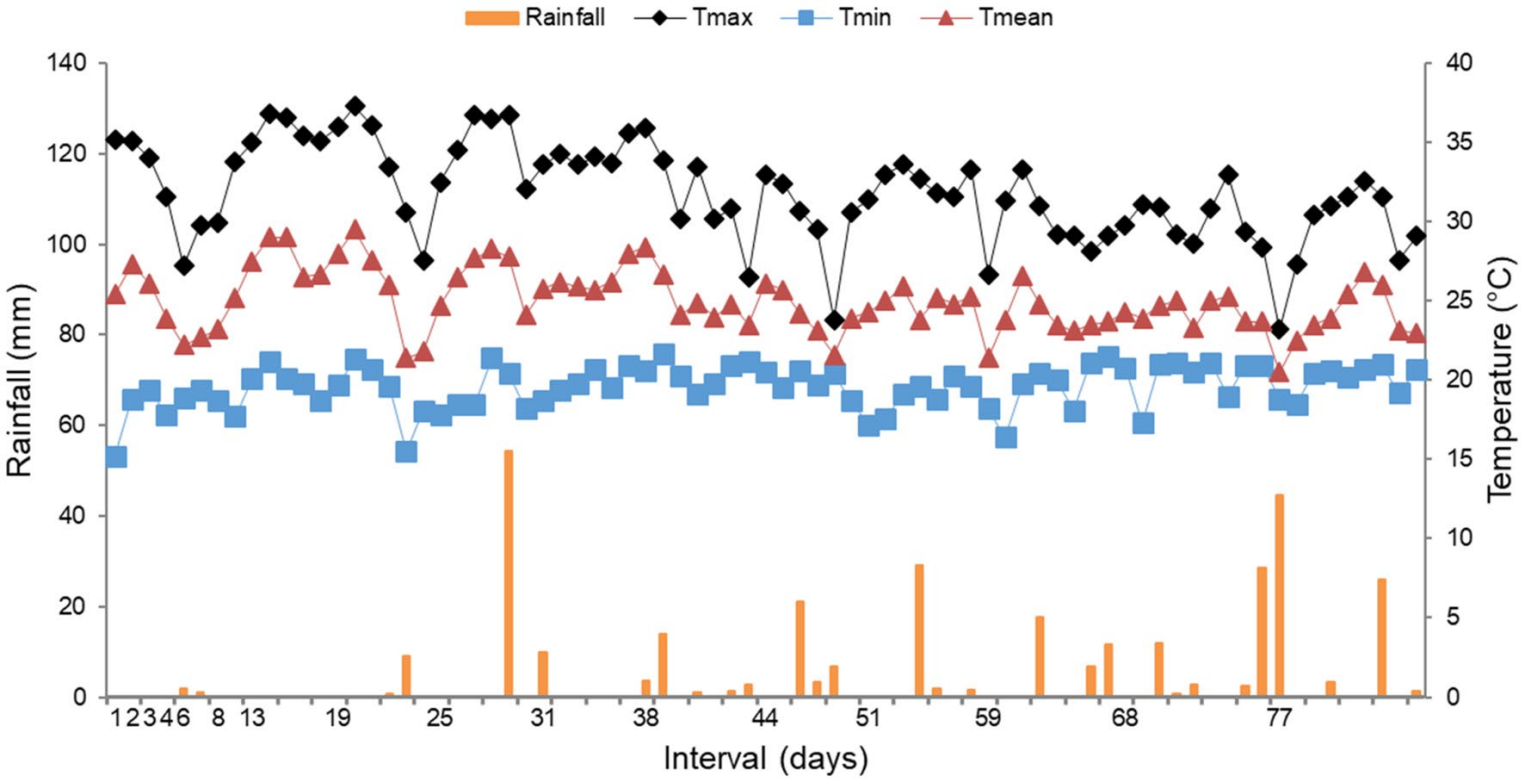
Daily rainfall and daily minimum (Tmin), daily mean (Tmean) and daily maximum (Tmax) ambient temperature throughout the NH_3_ emission sampling period. Data were retrieved from the Agroclimatological Station, Department of Exact Sciences, (FCAV/UNESP), located at 1 km from the experimental area.

Mean temperature during the NH_3_ emission sampling period was 25 °C. The lowest (15.2 °C) and highest (37.3 °C) temperatures were recorded on the first sampling day and on day 19, respectively. Cumulative precipitation throughout the experimental period was 320.5 mm, occurring on 30 different days (Fig. 4).

**Figure 5:**
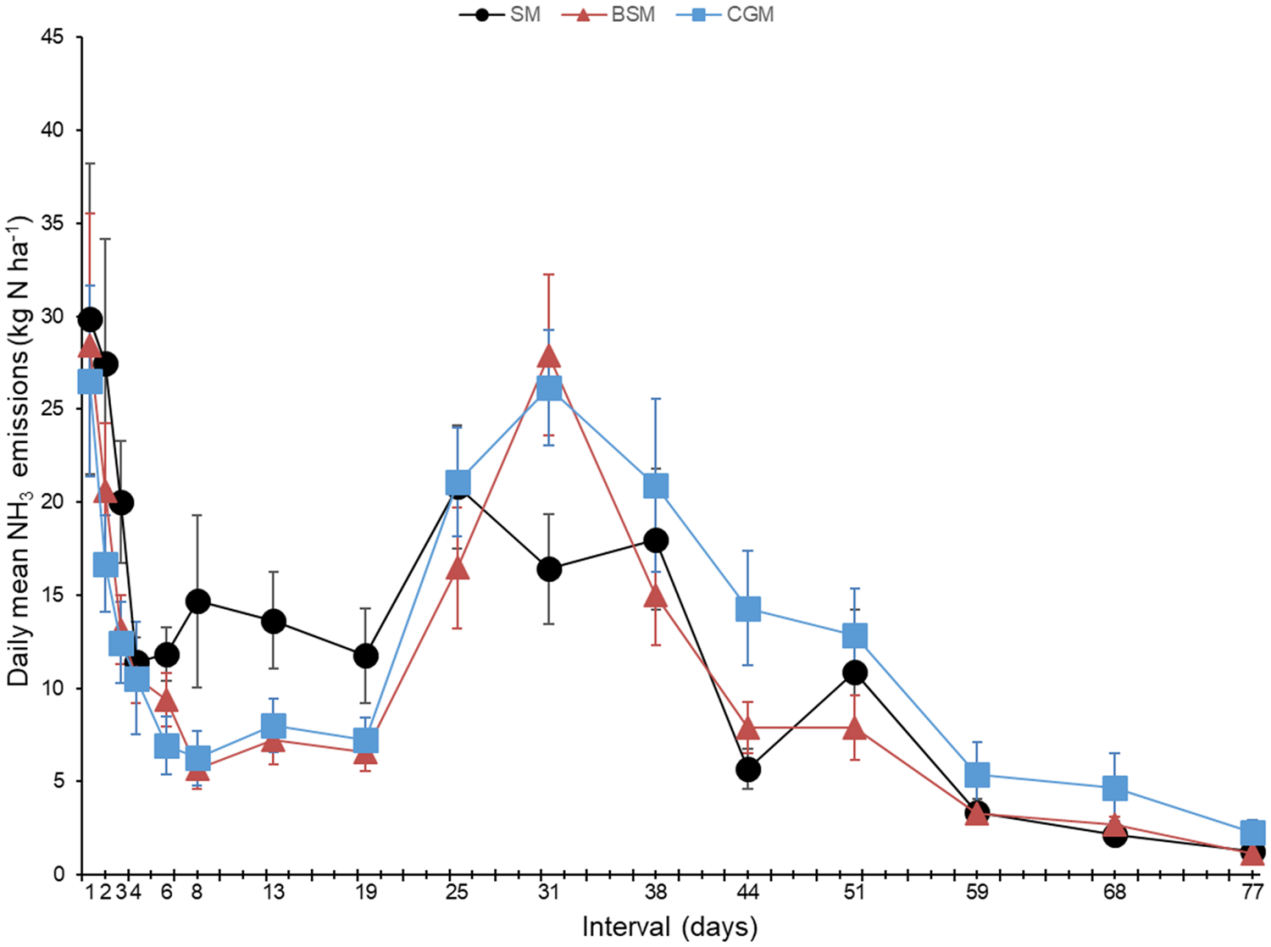
Daily mean NH_3_ emission from the manure of Nellore cattle fed with sources of rumen undegradable protein during the finishing phase in feedlot. Evaluations started after the animals were removed from the feedlot. SM = manure of animals fed soybean meal as a source of rumen degradable protein (RDP), BSM = manure of animals fed by-pass soybean meal as a source of rumen undegradable protein (RUP), CGM = manure of animals fed corn gluten meal as a source of RUP. Chamber considered as an experimental unit (n = 9). The error bars representing standard error of the mean.

Manure from all treatments showed the highest daily mean NH_3_ emission on the first day of evaluation (Fig. 5). Subsequently, NH_3_ emission decreased until the fourth day of evaluation under all treatments. From the 19th day, a new peak of NH_3_ emission was observed under all treatments. The SM treatment presented a small increase in NH_3_ emission on days 38 and 51, while the BSM and CGM treatments presented a decrease in emission. Ammonia emission under all treatments completely ceased on the 77th day. From day 13 to 25, cumulative NH_3_ emission under the SM treatment was higher than that under the BSM and CGM treatments. However, after this period, no differences were observed among the treatments.

There were no significant differences in cumulative NH_3_ emission from the manure during the evaluated period and manure content of DM, OM, N, and C (P > 0.05) among the three protein sources (Table 6). Likewise, there were no differences in the C/N ratio of the manure between the RDP and RUP sources (P = 0.491). However, the manure of animals fed BSM showed a higher C/N ratio than that of animals fed CGM (P < 0.001). The manure of animals fed RDP showed a higher NH_4_^+^ concentration than that of animals fed RUP (P < 0.001); however, there were no differences in NH_4_^+^ concentration between the manure of animals fed CGM and BSM (P = 0.670).

**Table 6:** Cumulative NH_3_ emission and manure characteristics of Nellore cattle fed with sources of rumen undegradable protein during the finishing phase in feedlot.

Variables^1^	Treatment^2^			SEM^3^	*p* value	
	SM	BSM	CGM		RDP vs*.* RUP	BSM vs*.* CGM
NH_3_, kg ha^−1^	800	707	882	112	0.687	0.259
DM, g kg^−1^ DM	913	800	827	59	0.231	0.312
OM, g kg^−1^ DM	565	618	579	24	0.475	0.197
N, g kg^−1^ DM	24.4	24.9	24.7	0.4	0.432	0.77
C, g kg^−1^ DM	271	309	287	14	0.185	0.163
C/N	11.1	11.3	10.7	0.1	0.487	< 0.001
NH_4_^+^, mg kg^−1^ DM	276	188	180	13	< 0.001	0.697

^1^NH_3_ = accumulated values during 77 days of evaluation. DM = dry matter, OM = organic matter; ^2^SM = manure of animals fed soybean meal as a source of rumen degradable protein (RDP), BSM = manure of animals fed by-pass soybean meal as a source of rumen undegradable protein (RUP), CGM = manure of animals fed corn gluten meal as a source of RUP. ^3^SEM = standard error of the mean. Chamber considered as an experimental unit (n = 9).

## Discussion

### Gas emissions

The use of RUP sources in the diet did not reduce N loss via urine. Meanwhile, it increased N_2_O emission but did not affect CH_4_ emission from manure. Therefore, our hypothesis that RUP inclusion in the diet would reduce N loss and contribute to reduced N_2_O and CH_4_ emissions from the manure was rejected.

The manure deposited in the soil enhances its N and C content, thereby altering the N mineralization rate and stimulating N_2_O production^29,30^. Meanwhile, labile C released during material decomposition regulates the seasonality of N_2_O and N_2_ production^30^. Inorganic forms of N (NH_4_^+^ and NO_3_^−^) are determinants of N_2_O production. The manure of animals fed BSM and CGM presented a higher NH_4_^+^ concentration than that of animals fed SM in the 42nd day, and in 63rd only for the CGM treatment, evidencing a temporal variation of the manure characteristics in relation to treatments, probably resulting in higher urea hydrolysis at these moments (Table 3, Fig. 3). The higher NH_4_^+^ concentration under the RUP treatments may have promoted nitrification or denitrification, resulting in N_2_O production^17^ (Table 2).

A reduction in urinary N was expected with the inclusion of RUP in diets, since an increase in N use efficiency is observed when lower RDP amounts are used in the diet^31^. In other words, reduced NH_3_ concentration in the rumen was compensated via increased urea recycling to maintain an optimum ruminal NH_3_ concentration for maximum microbial growth, thus decreasing its N urinary excretion^27,34,35^. However, this did not occur because the content of RDP in diets with RUP likely met the microbial demand for N, thereby not achieving sufficient urea recycling and allowing urinary N excretion similar to the diet with RDP (Table 1).

Elevated amounts of amino acids reaching the small intestine is another factor contributing to a greater N loss. When absorbed in excess or in imbalance relative to the animal’s requirements, these amino acids can be oxidized for energy production, leading to urea production in the liver, which is then excreted via urine. This might occur when the diet offers adequate levels of NH_3_ to meet the ruminal demand^34^. Therefore, excess CP concentration in the diet, either as RDP or RUP, may contribute to urinary N excretion.

The greatest N_2_O emission from the manure of animals fed the RUP sources (Table 3). This indicates that these diets probably had a higher urea content of the manure, since N_2_O emission is particularly affected by the urinary urea content^35^.

When higher RUP levels are used in the diet, a change in the route of urine-to-feces excretion is expected due to a higher amount of intact protein that reaches the intestine, which contributes to fecal N excretion when not absorbed^38,39^. However, there were no differences in fecal N excretion between the RDP and RUP treatments (Table 1), although fecal N concentration differed between the two RUP sources. This might be attributed to the distinct amino acid composition or the different chemical structures of these sources. The processes through which corn (corn gluten, a by-product of wet corn milling) and soybean (thermally treated) have been subjected can make the protein undegradable in the rumen or unavailable^38^.

Despite different compositions of the manure among the treatments (Table 3), there were no differences in CH_4_ emission (Table 2). Nitrogen and OM contents and C/N ratio of manure are important factors associated with CH_4_ emission^41,42^. Nevertheless, differences in manure chemical composition among the treatments were observed in some sampling days (Fig. 3). This result can be related to variations in environmental conditions, such as temperature and precipitation, which can alter the chemical composition of manure. However, these differences among the treatments were not consistent throughout the experimental period, justifying the lack of differences in CH_4_ emission.

In manure, most of the N content comes from N excreted via urine in the form of urea, which is rapidly hydrolyzed to NH_4_^+^, and N losses from organic forms of feces also occur^41^. Organic N can promote CH_4_ emission, playing an important role in the transformation of acetate to CH_4_^42^, whereas mineral N as NH_4_^+^ can inhibit CH_4_ production, breaking the link between acidification and methanogenesis in anaerobic processes^43^.

Nitrous oxide and CH_4_ fluxes varied from −62 to 318 µg N_2_O m^2^ h^−1^ and from −125 to 321 µg CH_4_ m^2^ h^−1^, respectively, during the experimental period (Fig. 2). These fluxes showed a great variation, which can be attributed to several factors, such as the temporal variation in the chemical composition of manure due to variations in climatic conditions, as explained above (Table 3, Fig. 3). Other researchers^44^ have reported a large variation in emissions, mainly associated with irregular fecal and urine deposition on the surface, which may also have occurred in the present study.

Frequent deposition and accumulation of feces and urine in the soil did not increase CH_4_ and N_2_O emissions over time under all treatments. Trampling by animals may have caused aeration of the surface material and have provided unfavorable environment for the action of methanogenic bacteria and nitrifying/denitrifying microorganisms. In addition, the humidity in the feedlot did not increase over time, based on the DM content of the manure, except on rainy days (Table 3). This is probably related to the dry climate at that time of year, associated with the compacted soil of the feedlot.

Precipitation and temperature changes strongly affect CH_4_ emission^45^. During the study period, CH_4_ flux was related to these variables. On the 21th day, increased emission peaks were observed under all treatments, probably due to precipitation in the previous week. Considering that CH_4_ emission occurs under anaerobic conditions, precipitation may have favored higher emissions due to increased moisture content of the manure^46^. On the 49th day, reduced CH_4_ emission was observed, possibly due to temperature drop on that day. Considering that CH_4_ emission is a biological and anaerobic process, temperature can act as a limiting factor by reducing methanogen activity^47^. After this period, CH_4_ emission tended to stabilize, probably due to the absence of high precipitation and little variation in temperature (Fig. 1).

The mean CH_4_ emission under all treatments during the finishing phase in the feedlot (SM = 53 µg C–CH_4_ m^2^ h^−1^; BSM = 33 µg C–CH_4_ m^2^ h^−1^; CGM = 16 µg C–CH_4_ m^2^ h^−1^; mean of 8.8 g C–CH_4_ day^−1^ pen^−1^) in the present study was lower than reported values by other researchers (mean of 110 g day^−1^ pen^−1^)^48^ under similar climatic conditions and a pen density of 6 m^2^ per animal, however, the floor was concreted and the excreta were removed every 15 days. The low moisture of the manure was possibly responsible for low CH_4_ emissions, because even under favorable chemical conditions, microbial activity is limited at low moisture levels. Of note, the density in each pen was 30 m^2^ per animal and the evaluations were performed near the feeders, in an area of 6.5 m × 10 m with higher excreta deposition. The density of animals is reflected in the condition of excreta deposition and accumulation on the surface, and it is a relevant factor to be considered when evaluating gas emissions in feedlots^49^.

On some sampling days, CH_4_ uptake occurred predominantly through the consumption of atmospheric CH_4_, which can occur in aerobic environments^39^. The environment is a CH_4_ source when the balance between methanogenic production and methanotrophic consumption is positive, leading to CH_4_ emission. In contrast, when this balance is negative, the environment is considered a CH_4_ sink^39^.

Considering that the feedlot system has emerged as a management strategy to minimize the impacts of lower forage production in the dry season, majority of feedlots in Brazil are managed from April to November, when rainfall is scarce and temperature is low. The climatic conditions during this period, when associated with feedlots of low animal density, can result in low CH_4_ emission. In an inventory to estimate GHG emission in Brazil^50^, it is clear that we do not have enough data to estimate emissions from Brazilian feedlots. Therefore, measurements must cover different systems, with different stockings, feedings and manure management to generate concrete data that allow the comparison between mitigation strategies.

The mean N_2_O fluxes (SM = 22 µg N–N_2_O m^2^ h^−1^; BSM = 59 µg N–N_2_O m^2^ h^−1^; CGM = 36 µg N–N_2_O m^2^ h^−1^; 12 g N–N_2_O day^−1^ pen^−1^) observed in the present study were high than to some report values reported ( 0.8 g N–N_2_O day^−1^ pen^−1^)^48^ even considering a higher density (6 m^2^ animal^−1^) and removal of excreta from the area every 15 days. A higher peak of N_2_O emission was observed on the 21st day under all treatments, possibly due to rainfall in the previous week. Other researchers^51^ in a controlled experiment simulating open feedlot, demonstrate increased emissions following precipitation events, with peaks that vary 2 h to 15 days after the rain.

Post-rainfall emissions and wetting of the area might be related to a combination of mineralization, nitrification, and/or denitrification, leading to the release of N_2_O absorbed in the dry soil^52^. Moisture is an important factor in N_2_O production, particularly when associated with temperature and a propitious chemical composition^53^, emission of N_2_O increases markedly with increasing temperature^54^. However, after reaching the peak, N_2_O emissions remained stable, with small variations across evaluation days; even in the presence of additional precipitation events, low temperature (minimum of 3.3 °C near the 49st day) may have hampered the occurrence of new emission peaks.

Nitrate was not detectable in the manure during the experiment. Nitrous oxide production is assumed to occur through nitrification, via the oxidation of NH_4_^+^ in hydroxylamine (NH_2_OH), with NOH as an intermediate and N_2_O as the product^55^. N_2_O can also be produced through denitrification by nitrifiers, wherein NH_3_ is nitrified and oxidized to nitrite (NO_2_^−^), which is then reduced to nitric oxide (NO), N_2_O, and molecular N (N_2_). Nitrous oxide is an intermediate in the reduction of NO_2_^−^ to N_2_^56^. During denitrification, NO_3_^−^ is used as the primary substrate^57^. Denitrification may not have occurred in the present study.

Correlation analyses showed no significant linear associations of CH_4_ and N_2_O production with the tested variables related to the chemical composition of manure, which can be attributed to specific factors (Tables 4 and 5). The processes underlying the production of gases are complex and rely on the chemical composition of manure. In addition to the chemical composition, the emission of gases in the manure is dependent on other factors such as temperature, moisture, deposition conditions, and trampling by animals. The absence of significant correlations between gas production and manure composition may be related to the small variation in the characteristics analyzed during the sampling period, making it difficult to observe relationships among variables.

### NH_3_ emission

The use of RUP in the diet did not reduce N loss via urine and did not influence NH_3_ emission from the manure. In this sense, our hypothesis that RUP inclusion in the diet would reduce N loss and contribute to decreased NH_3_ emission was rejected.

The manure of animals fed SM presented higher NH_3_ emissions than that of animals fed CGM and BSM from the 8th to 25th day of evaluation. This may be attributed to the higher NH_4_^+^ content of the manure of animals fed SM than that of animals fed CGM and BSM at the beginning of the sampling period (Table 6). Subsequently, the manure of animals fed CGM and BSM presented a new NH_3_ emission peak following the event of the highest precipitation (54.2 mm) throughout the experimental period. However, during this period, most of the NH_4_^+^ from the SM treatment had already been used, as reflected by the weak response to precipitation under this treatment. Urea present in the urine and feces is rapidly hydrolyzed, and the formed NH_4_^+^ is dissociated to aqueous NH_3_, depending on NH_4_^+^ concentration and pH of manure and environmental conditions. When precipitation occurs, urease activity is promoted, resulting in increased NH_3_ emission^58^. Of note, however, manure sampling for characterization was performed before implanting the chambers in the area. Thus, the chemical composition data presented herein do not represent the possible temporal variations during the NH_3_ evaluation period (Table 3).

Higher values of NH_3_ emission have been reported (49.1 kg NH_3_ animal^−1^) in beef cattle feedlots, which is mainly related to the fact that the majority of confinement feedlots are outdoors, given that wind speed in open environments increases emission^59^. According to others studies^19^, daily NH_3_ emission in feedlots rarely exceeds 2000 µg NH_3_ m^−3^; however, in the present study, higher values were observed. Importantly, as explained before, the evaluations were performed in an area of higher excreta deposition, with the objective of comparing the treatments in homogeneous conditions of excreta distribution. Therefore, the amount of emission by area of the total feedlot may have been overestimated in this study. Conversely, we did not account for emissions when the animals were present in the feedlots. Throughout the sampling period, the animals had already been removed from the area, and there was a large amount of accumulated manure. When the wet season starts, emission may have been favoured by increased moisture content due to the large amount of available substrate^19^. Therefore, the urea excreted by the animals was hydrolyzed and contributed to the stock of NH_4_^+^, which was emitted as NH_3_ when the moisture content increased as a function of precipitation.

Over time, as no new manure was deposited due to the absence of animals in the area, emission probably ceased when the substrate was consumed, which occurred around the 77th day in the present study. In experiments in which excreta from the animals is collected and then applied to the soil for evaluation in the absence of animals and new depositions, ammonia emission occurs for 3 weeks on average^42,62,63^. Therefore, further studies are warranted to investigate NH_3_ emission in open feedlots and to observe peaks occurrence in the presence of animals, maintaining the evaluations also after removing the animals, in the next rainy season.

## Conclusions

The inclusion of RUP in the diet did not affect N excretion by animals. While the N_2_O emission from the manure was increased, CH_4_ emission and NH_3_ emission remained unaffected. Additional studies are warranted to investigate the effects of using different proportions of RDP and RUP in diets on NH_3_, N_2_O, and CH_4_ emissions from the manure of animals managed in feedlot systems under tropical conditions.

## Material and methods

The experiment was approved by the Ethics, Bioethics, and Animal Welfare Committee of São Paulo State University (UNESP), Jaboticabal, under protocol numbered 16.668/16. All methods were carried out in accordance with relevant guidelines and regulations. Methods are reported in the manuscript following the recommendations in the ARRIVE guidelines.

### Site description

The present study was conducted at the Campus of Jaboticabal of the São Paulo State University, Sao Paulo, Brazil (21°14′05″S, 48°17′09″W; altitude, 615.01 m). The region has a tropical climate, with a dry season from April to September and a wet season from October to March, during which over 80% of the annual precipitation occurs. The soil is Rhodic Ferralsol^62^ derived from basalt, with a sandy–clay–loam texture (10% silt and 61% sand) in the surface layer (0–10 cm). The soil pH in CaCl_2_ is 5.9, bulk density is 1.8 kg dm^−3^, and organic matter content is 16.6 g dm^−3^ at the same depth.

Meteorological data (daily precipitation and ambient temperature) were obtained from the dataset of the Agrometeorological Station of the Department of Exact Sciences, Universidade Estadual Paulista (UNESP), Campus of Jaboticabal, located 1 km from the experimental area.

### Experimental design

The experiment was conducted for 210 days from May to December 2019. The first 21 days were dedicated to animal adaptation to the diet, followed by 112 days of confinement, during which weekly sampling of N_2_O and CH_4_ was performed. After removing the animals from the feedlots, NH_3_ was sampled for 77 days.

Fifty-four Nellore bulls with an initial body weight of approximately 360 kg were distributed in three treatments. The animals were divided into three treatments and allocated in collective pens (11 m × 50 m; one pen per treatment and 18 animals per pen). Each pen had a dirt floor with collective drinkers for every two pens. There were two covered automated feeders in each pen (INTERGADO®, Intergado Ltd., Contagem, Minas Gerais, Brazil). The feed system was equipped with an automated feeder monitor resting on load cells, allowing electronic registration of the amount of feed consumed by animal. The trough recognizes the animal from the electronic ear tag, automatically sends consumption data to a database, and stores the information.

Manure of animals fed with sources of protein (two sources of RUP and one source of RDP as a control) was collected, resulting in three treatments as follows:

1. Soybean meal (SM): source of RDP.
2. By-pass soybean meal (BSM): source of RUP
3. Corn gluten meal (CGM): source of RUP.

The experimental diets were composed of 30% roughage and 70% concentrate, formulated to meet the average daily gain (ADG) of 1.5 kg day^−1^, according to BR CORTE^63^. The diets were offered at 08:00 am and 04:00 pm. The amounts offered were sufficient to allow a daily leftover of 5–10% of the total offered.

The ingredients of the diets were analyz ed for chemical composition (Table 7). The AOAC^64^ method was used to determine dry matter (DM) (method 930.15), crude protein (CP) (method 990.03), organic matter (OM) (method 942.05), and ether extract (EE) (method 920.39) content. Neutral detergent fiber (NDF) content was determined according to the method described by^65^ using ANKOM® 2000 (Ankom Technologies, New York, USA) with thermostable α-amylase and without sodium sulfite, corrected for ashes and residual proteins. The RDP and RUP content was estimated based on the protein fraction^66^ and degradation rate of each fraction, considering a passage rate of 5% h^−1^.

**Table 7:** Ingredients and chemical composition of the diets.

Diet composition, g kg^−1^ DM	Diets^1^		
	SM	BSM	CGM
Corn silage	300.2	299.7	301.5
Ground corn	134.6	134.4	134.2
Citric pulp	383.0	397.5	421.6
Soybean meal	172.7	–	–
By-pass soybean meal	–	159.0	–
Corn gluten meal	–	–	132.3
Mineral mix	9.4	9.4	10.4
**Chemical composition**^**2**^			
Dry matter, g kg^−1^ as fed	538.1	538.4	537.2
Organic matter, g kg^−1^ DM	918.9	917.9	925.1
Total digestible nutrients, g kg^−1^ DM	745.7	741.3	751.3
Crude protein, g kg^−1^ DM	163.4	153.6	164.4
RDP, g kg^−1^ CP	665.0	494.1	446.3
RUP, g kg^−1^ CP	335.0	505.9	553.6
Neutral detergent fiber, g kg^−1^ DM	301.9	321.5	302.5
Ether extract, g kg^−1^ DM	39.5	40.0	39.2
Non-fibrous carbohydrates, g kg^−1^ DM	414.2	402.8	419.0

^1^SM = manure of animals fed soybean meal as a source of rumen degradable protein (RDP), BSM = manure of animals fed by-pass soybean meal as a source of rumen undegradable protein (RUP), CGM = manure of animals fed corn gluten meal as a source of RUP; ^2^DM = dry matter, CP = crude protein.

**Figure 6:**
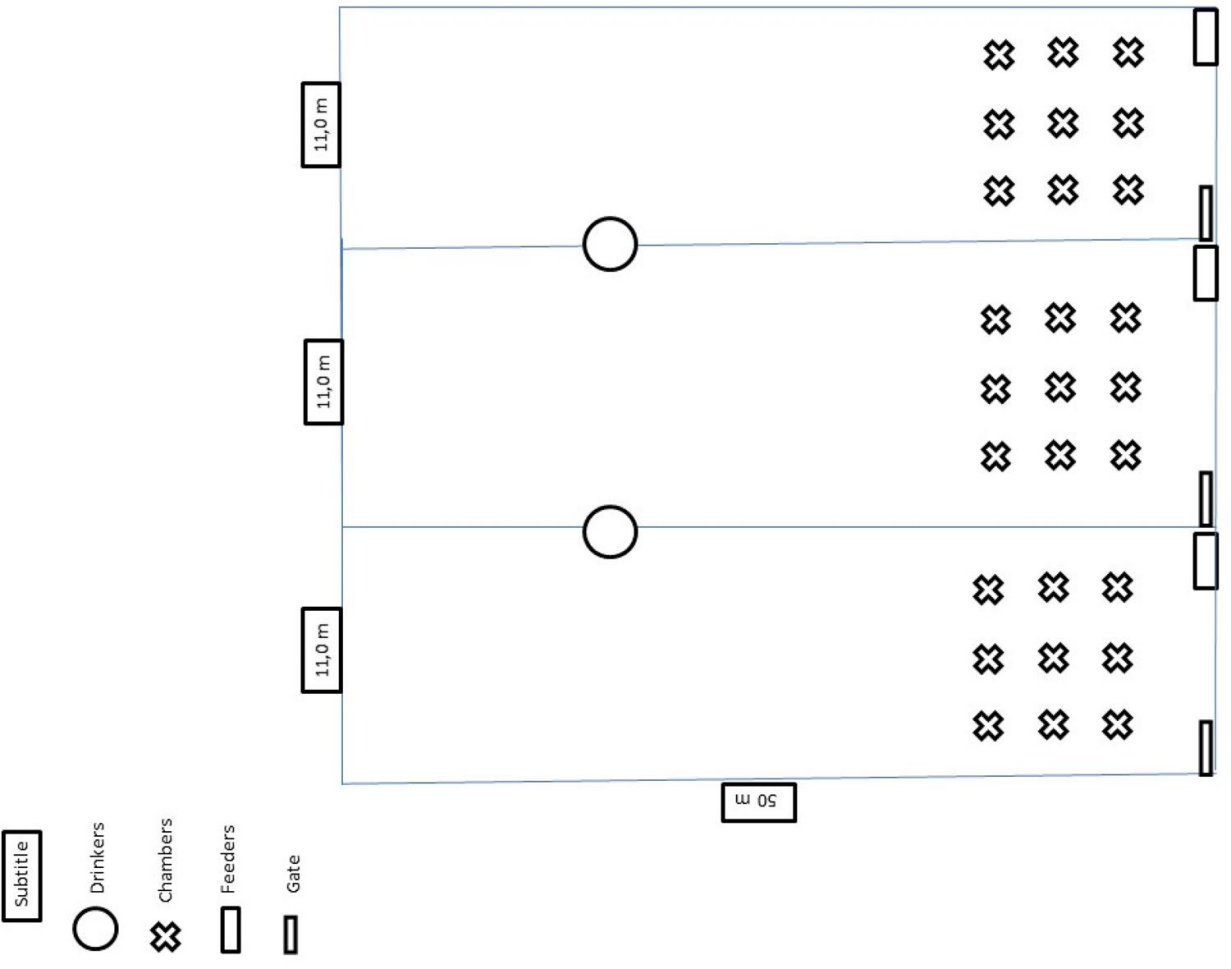
Map of the experimental area.

Gases (N_2_O, CH_4_ and NH_3_) were sampled using chambers (n = 9 per treatment) arranged in an area of 65 m^2^, near the feeders, where the manure (feces and urine) was deposited the most frequently. The chambers were placed on manure (feces and urine) that had been deposited on the feedlot surface by animals subjected to treatments. At the time of evaluation, the chambers were randomly placed in an area (6.5 m × 10 m) delimited near the feeders inside each confinement pen. Specifically, an area of higher excreta deposition was selected with the objective of treatment comparison, thus avoiding evaluation in places without homogenous excreta distribution (Fig. 6).

### Evaluation of N_2_O and CH_4_ emissions

Nitrous oxide and CH_4_ emissions were determined using static closed chambers, according to the recommendations of the manual for GHG evaluation^67^. Plastic chambers (0.6 m × 0.4 m × 0.24 m) coated with a thermal insulator were positioned above the manure only at the time of gas collection, allowing the animals to trample, defecate and urinate freely around in the area. Sampling was performed once a week throughout the feedlot period (112 days), totaling 16 sampling events. Sampling was carried out between 4:00 pm and 04:00 pm. The chambers were closed for 20 min, and air samples were collected at 0, 10, and 20 min using a 50 mL polypropylene syringe and then transferred to previously evacuated chromatography flasks (20 mL). The temperature inside and outside the chamber was measured using a digital thermometer (Incoterm®) to correct gas fluxes.

Air samples were analyzed using gas chromatography (Shimadzu Greenhouse Gas Analyzer GC-2014; Kyoto, Japan) under the following conditions: (1) N_2_O: injector temperature, 250 °C; column temperature, 80 °C; N_2_ carrier gas (30 mL min^−1^); and electron capture detector temperature, 325 °C; and (2) CH_4_: H_2_ flame gas (30 mL min^−1^) and flame ingestion detector temperature, 280 °C.

Nitrous oxide (µg N–N_2_O m^−2^ h^−1^) and CH_4_ (µg C–CH_4_ m^−2^ h^−1^) fluxes were calculated considering a linear increase in gas concentration inside the chamber during the closed period and corrected for ambient temperature, ambient pressure, and chamber dimensions, as follows:

$$ {\text{Gas}}\;{\text{flow}} = ({\text{gas}} \times {\text{M}}\omega \times {\text{V}} \times {6}0 \times {1}0^{{ - {6}}} )/{\text{A}} \times {\text{VM}}_{{{\text{corr}}}} \times {1}0^{{ - {9}}} ) $$

where *gas* is the increment in the gas concentration inside the chamber during the closed period (ppb min^−1^); *Mω* is the molar mass of N–N_2_O (28 g mol^−1^) or C–CH_4_ (12 g mol^−1^); *V* is the chamber volume (m^3^); 60 is the conversion factor from minutes to hours; 10^–6^ represents the conversion factor from g to µg; *A* is the chamber area (m^2^); *VM*_*corr*_ is the molecular volume corrected by the normal conditions of temperature and pressure at the time of sampling; and 10^–9^ is the conversion factor from ppb to µL m^−3^.

Fluxes were multiplied by 24 to obtain daily emissions, and the daily values were integrated through linear interpolation to obtain cumulative emissions during the evaluated period. Negative fluxes were included in the calculations to avoid biased data^68^.

### Evaluation of NH_3_ emission

After removing the animals from feedlots, NH_3_ volatilization was evaluated until the NH_3_ emission ceased by sampling on days 1, 2, 3, 4, 6, 8, 13, 19, 25, 31, 38, 44, 51, 59, 68 and 77 after positioning the chamber. The chambers were randomly placed above the manure (feces and urine) in the previously delimited areas. Quantification was performed according to the methodology of static chamber^69^, using semi-open chambers made of plastic bottles containing a foam soaked in 10 mL of 1.0 mol dm^−3^ H_2_SO_4_ solution + glycerin 2% (v/v) to capture N. The amount of N-NH_3_ retained in the foam was determined by distillation, following the Kjeldhal method (method 973.49)^61^ and a correction factor of 1.74 was used^69^.

### Manure analysis

Manure samples composed of feces and urine deposited in the feedlot surface material soil, trampled by the animals, were collected on days 42, 63 and 105 after N_2_O and CH_4_ evaluations, directly above the ground surface at the places where the chambers were positioned. The samples were analyzed for DM (method 930.15)^64^, OM (method 942.05)^64^, total C, total N (dry combustion method, using Leco® CN-828, Leco Corporation, Michigan, USA), and soil inorganic N (NO_3_^−^ and NH_4_^+^) (distillation using magnesium oxide and Devarda’s alloy, method 973.49)^64^ content.

### Estimation of fecal and urinary production and N balance

Fecal production was estimated using the internal marker technique^70^ based on the indigestible NDF (NDFi) marker. Fecal sampling was performed from the 60th day after the animals entered the feedlots, for three consecutive days, directly from the rectum of the animals. Sampling was performed in the morning, middle of the day, and afternoon on the first, second, and third days, respectively. A composite fecal sample, by animal (9 animals/treatment), were made with the samples from these three days. The samples were mixed, homogenized, partially dried in a forced-air ventilation oven at 55 °C for 72 h, and milled in a mill with a 2 mm sieve. Samples of the ingredients of the animals’ diets were collected, and their consumption was determined using the INTERGADO®.

Fecal NDFi content was determined after incubating the samples in situ for 288 h^71^ followed by extraction with neutral detergent using an autoclave^72^. Fecal DM production was determined as the ratio of the concentration of the internal indicator ingested by the animal and its concentration in feces^73^.

Urine samples were collected simultaneously with fecal samples. In brief, 50 mL aliquots of urine were sampled (“*spot*” sample) during three consecutive days^74^. Creatinine concentration in the spot sample was determined with a colorimetric method using a commercial kit (Labtest®). Urinary excretion was estimated based on the association between creatinine excretion and body weight using the equation proposed by^75^:

$$ {\text{UCE}}\left( {{\text{g day}}^{{ - {1}}} } \right) = 0.0{345 } \times {\text{ BW}}^{{0.{9491}}} $$

where UCE = urinary creatinine excretion and BW = body weight in kg.

The fecal and urine samples were analyzed for total C and total N content using the dry combustion method with Leco® CN-828 (Leco Corporation). Nitrogen retention (NR) was expressed in grams per day and in percentage of NC, and fecal and urinary N excretion was expressed as the percentage of the total material excreted. The following equation was used to calculate NR:

$$ {\text{NR}} = {\text{NC}}\left( {{\text{g day}}^{{ - {1}}} } \right) - \left[ {{\text{EFN }}\left( {{\text{g day}}^{{ - {1}}} } \right) + {\text{EUN }}\left( {{\text{g day}}^{{ - {1}}} } \right)} \right] $$

where NC = N consumption, EFN = excretion of fecal N, and EUN = excretion of urinary N.

### Statistics

All statistical analyses were performed using SAS 9.4 (SAS Inc., Cary, NC). Response variables were analyzed in a completely randomized design using the PROC MIXED procedure. There were nine experimental units per treatment. Mean values were compared using orthogonal contrasts (SM *vs.* RUP and BSM *vs.* CGM) at a 5% probability level.

Total N, total C, and C/N in feces and urine and N balance were analyzed considering a model including the treatments (SM, BSM, and CGM) as fixed effects, animals (experimental unit in the RANDOM SAS option) and residual random error (NIID) of (0, σ^2^) as random effects.

Cumulative N_2_O, CH_4_ and NH_3_ emissions, and manure characteristics (DM, OM, N, C, C/N, NH_4_^+^, and NO_3_^−^ of manure, sampled on day 0, before the beginning of NH_3_ emissions measurements) were analyzed considering a model including the treatments (SM, BSM, and CGM) as fixed effects, chamber (experimental unit in the RANDOM SAS option) and residual random error (NIID) of (0, σ^2^) as random effects.

Nitrous oxide and CH_4_ daily fluxes and manure characteristics (DM, OM, N, C, C/N, NH_4_^+^, and NO_3_^−^, sampled on days 42, 63 and 105 of N_2_O and CH_4_ evaluation) were analyzed using a repeated measures mixed model over time including the treatments (SM, BSM, and CGM), collection period and interaction as fixed effects, chamber (experimental unit and RANDOM SAS option) and residual random error (NIID) of (0, σ^2^) as random effects. Distinct covariance matrices were evaluated and the best structure was selected according to the Akaike information criterion (AIC).

Pearson correlation analysis between gas emission (N_2_O and CH_4_) and chemical composition (N, C, C/N, DM, OM, and NH_4_^+^) of the manure was performed separately for each sampling day (days 42, 63 and 105 of manure evaluation), and also considering all data collected on these days.
